# Aging Into Disability: A Conceptual Challenge for Gerontology

**DOI:** 10.1093/geroni/igab046.2072

**Published:** 2021-12-17

**Authors:** Jeffrey Kahana, Timothy Goler, Lawrence Force

**Affiliations:** 1 Mount Saint Mary College, Newburgh, New York, United States; 2 Norfolk State University, Newport News, Virginia, United States; 3 Mount Saint Mary College / C enter on Aging and Disability Policy, Mount Saint Mary College, New York, United States

## Abstract

One of the most fraught subjects facing a fast growing aging population is the subject of aging into disability. This paper examines the processes of aging into disability as a distinct challenge for not only older persons, but also for the field of gerontology, and public policy-makers. Disability in youth and in middle age has largely defined the disability rights agenda, and elders aging into disability have not been the subject of much attention from scholars in the field of disability. Surprisingly, however, scholars and policy-makers in gerontology have also by and large avoided the subject of older persons aging into disability—a complex process that involves impairment, environmental disablement, and changes in social relationships. This process accelerates with advancing age, and disproportionately affects women. Moreover, when older adults develop mobility limitations, experience falls, become hard of hearing, or experience other such impairments of age related disability, they do not think of themselves as aging into disability, or being disabled. This lack of disability identity may protect them from stigma and from low self-esteem. At the same time, it stands in the way of seeking accommodations and from developing a bond with other older adults who are aging into disability. This paper explores the dynamics of disability avoidance as an ideal that can harm older adults and their caregivers. It aims to bring disability more fully into the normal life-course, and to suggest lines of inquiry for gerontological research, to broaden the field, and to make service communities more inclusive .

